# Advancing type 1 diabetes therapy: autologous islet transplant breakthrough

**DOI:** 10.1038/s41392-024-02090-x

**Published:** 2024-12-23

**Authors:** Ram Abou Zaki, Assam El-Osta

**Affiliations:** 1 Baker Heart and Diabetes Institute, Epigenetics in Human Health and Disease Program, Melbourne, VIC Australia; 2 Baker Department of Cardiometabolic Health, The University of Melbourne, Parkville, VIC Australia; 3 School of Translational Medicine, Department of Diabetes, Monash University, Melbourne, VIC Australia; 4 Department of Medicine and Therapeutics, The Chinese University of Hong Kong (CUHK), Hong Kong SAR, China

**Keywords:** Mesenchymal stem cells, Stem-cell research

The first patient to receive autologous transplantation of chemically induced pluripotent stem-cell-derived islets (CiPSC islets) was recently announced in the journal *Cell*. The breakthrough surgery by Wang and colleagues at Nankai University, achieved year-long insulin independence for an adult living with type 1 diabetes (T1D).^1^

While current pharmaceutical options for diabetes management help control blood glucose levels they do not prevent, retard or reverse the decline in insulin-secreting β-cells. Consequently, regenerative medicine has focussed on β-cell replacement which remains an unmet medical need. In constructing islet organoids in the adult mouse, scientists identified resident protein C receptor-positive progenitors that can expand to resemble β, α, δ and PP cells to form glucose-responsive islet-like organoids that secrete insulin.^2^ Indeed, long term expansion of these islet organoids could reverse diabetes in mouse models. While translating preclinical therapies using animal models can be difficult, a recent Phase 1/2 study (NCT03163511) using human pluripotent stem cell-derived pancreatic endodermal cells showed β-cell maturity could restore insulin levels in T1D patients. While these experiments raised the benefit of cell replacement therapy, the study fell short of long-term insulin independence. Earlier this year, a Shanghai transplantation group carried out further studies by treating a T2D patient with impaired pancreatic function using endodermal stem cell derived islets.^3^ The islets were well tolerated without immune rejection, improved glycaemic control and reduced insulin dependence.

In this context of significant technological advancements in regenerative science, the study by Wang and colleagues is a breakthrough moment in medicine.^1^ The transplant recipient was a 25-year-old female living with T1D that achieved stable glycaemic control following surgery and without complications. The study is novel for different reasons. First, the clinical procedure involved autologous graft surgery that was intent on side-stepping immune rejection by means of chemically reprogramming the patient’s own adipose tissue. This surgical paradigm also ushers in a new era for transplantation, one that no longer relies on donors, effectively addressing the challenges posed by organ donor shortages. Second, the impressive yet demanding workflow involved multiple drugs to purposely reshape recipient adipose-derived mesenchymal stem cells to differentiate into functional insulin-producing islets. Third, and important surgically, transplantation was substantially less invasive because the graft was positioned beneath the abdominal rectus muscle. This cutting-edge approach not only simplified the surgical procedure and follow-up monitoring, but this program also ensured superior graft survival and maturation. Taken together, these advancements not only elevate the therapeutic potential for T1D but also herald a new era in regenerative medicine.

Medical evidence of long-term efficacy for any major technological advance is critical and the scientists showed the recipient’s insulin requirements had decreased by week two of engraftment, achieving complete insulin freedom at day 75 and that was preserved for no less than 12 months. Fasting blood glucose levels were also improved, dramatically shifting from 210 ± 59 mg/dL (before surgery) to 126 mg/dL (after surgery). Moreover, patient time-in-range was upturned from 43.18% to >98% during the eight months of follow-up, while HbA1c dropped from baseline levels of 7.57% to 5.37% by day 120. Importantly, all of these clinical signs were below the diagnostic threshold for diabetes.

While lifesaving transplantation has been considered for decades as a potential therapeutic solution for people living with T1D, the challenge for newly generated β-cells has been autoimmune rejection. The outcome of the CiPSC islet procedure was achieving fasting C-peptide levels that ranged between 407.1–721.6 pmol/L. Before engraftment C-peptide was undetectable in the recipient. Furthermore, the BETA-2 score, an indicator of β-cell graft function, was barely detectable at 0.13 before surgery and transformed to a composite score of 41.83 by day 365. Scientists have long experimented with techniques that achieve a score of >20 because this mirrors true functional recovery of the islet’s engraftment. A BETA-2 score of >30 indicates ideal function, with minimal need for exogenous insulin.

**Fig. 1:**
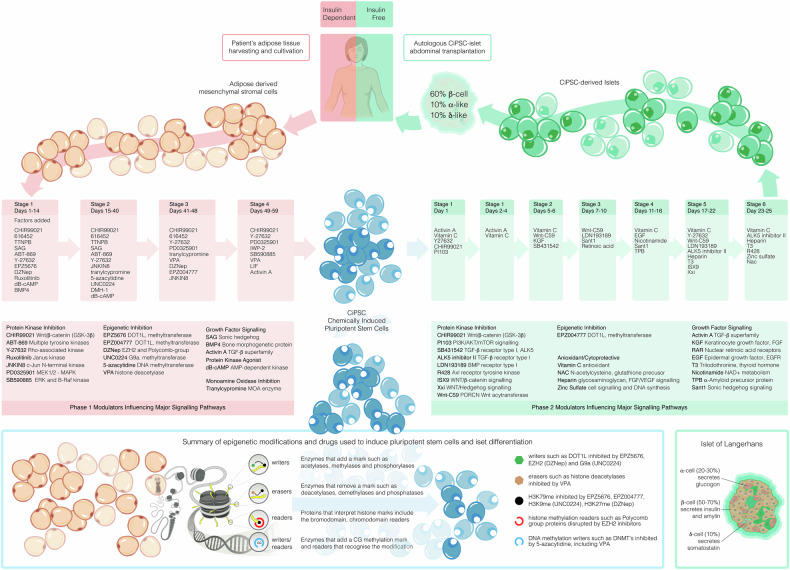
Adipose-derived mesenchymal stromal cells were isolated from a 25-year-old T1D patient and reprogrammed into chemically induced pluripotent stem cells (CiPSC’s) over a 59-day period employing a complex differentiation strategy of pharmacological stimulation designed in four discrete stages. Modulators influencing major signalling pathways were employed to manufacture patient-derived CiSPC-islets. The first category was the disruption of the signal transduction pathways of protein kinases by several modes of inhibition. CHIR99021, ABT869, Y27632, Ruxolitinib, JNKIN8, PD0325901, and SB590885 are signalling pathway inhibitors targeting key molecular pathways (Wnt/β-catenin, angiogenesis, ROCK, JAK-STAT, MAPK). Impressive advances were also made against core pathways involved in epigenetic regulation. EPZ5676 (Pinometostat) and EPZ004777 inhibit protein methyltransferase DOT1L, DZNep targets the inhibition of the EZH2 histone methyltransferase, UNC0224 inhibits histone methyltransferase G9a, 5-azacytidine promotes DNA hypomethylation by inhibiting DNA methyltransferase, and valproic acid (VPA) acts as a histone deacetylase inhibitor, promoting histone acetylation and indirectly influencing DNA demethylation. Pathways involved in growth factor signalling were also targeted using SAG (Smoothened Agonist), Bone Morphogenetic Protein 4 (BMP4) and Activin A which are part of the TGF-β superfamily and is involved in stem cell function. In the second phase, Activin A, KGF, retinoic acid, EGF, T3, and nicotinamide were added to regulate growth factor signalling and cell fate. The protein kinase inhibitors PI103, SB431542 and LDN193189 targeted key signalling pathways that influence stem cell differentiation. EPZ004777 inhibits protein methyltransferase DOT1L. Antioxidants were added and include Vitamin C, N-acetylcysteine (NAC), Heparin, and Zinc Sulfate protected cells from oxidative stress and promoted survival during differentiation. The small molecule TPB was used to modulate cellular behaviour. The islets comprised approxiamately of 60% β-cells, 10% α-cells and 10% δ-cells and were injected underneath the abdominal anterior rectus sheath of the patient who remained diabetes free at 1-year follow-up

At yearlong, recovery by the patient was not a dramatic sequence of lucky moments for the medical team, but rather, preceded by extensive workup and technological developments intended to optimise human protocols for stem cell reprogramming.^4^ The chemical approach to the regeneration of CiPSC islets used human adult adipose-derived mesenchymal stromal cells and human adult skin dermal fibroblasts. The reprogramming efficiency ranged from 0.21% to 2.56% when tested in eight independent donors. The Nankai researchers employed a multi-stage protocol using small molecules such as 5-azacytidine, tranylcypromine, DZNep, and EPZ004777 to influence *Oct4*, *Sox2* and *Nanog* activation and dedifferentiation of adipose cells into pluripotent stem cells (Fig. 1). The manufacturing process involved stepwise optimization with ISX9 and Wnt-C59 for the generation of islet-sized aggregates expressing NKX6.1+C-peptide+ cells at an efficiency of up to 70%. To harness the newly generated patient derived islets the scientists confirmed that Chromogranin A (*CHGA*) positive and Neurogenin 3 (*Ngn3*) negative endocrine cells were glucose-responsive and insulin secreting. The derived islets were also positive for the mature β-cell markers, *MafA* and *UCN3*, known regulators of ductal progenitor cell expansion. This protocol shows what can be done to harness stem cell pluripotency from the endodermal lineage. Recent studies have also demonstrated parallel activation of the proliferative *Ki67* marker alongside trajectorial commitment and expression of *Sox9* and *MafA* from pancreatic tissues derived from human donors following ex vivo stimulation with pharmacological EZH2 inhibitors.^5^ So effective were GSK126 and Tazemostat in influencing β-cell regenerative capacity, that the study of pancreatic ductal progenitor cells showed reduced H3K27me3 levels at the *Ins* (insulin) and *Igf2AS* genes. This process signifies epigenetic signalling as an important transcriptional step towards advancing differentiation capacity, β-cell regeneration and insulin function.

The patient’s medical history was relevant to the immunosuppressive drugs used to avoid long-term autoimmune rejection and islet destruction. While allografts from different donors can trigger immune rejection, autografts eliminate that risk. Very simply, the approach was clear, yet - the Nankai team understood that self-reactive T cells have a memory phenotype in T1D. Scientists have puzzled for years over autoimmune β-cell destruction, but the process of autoreactive T-cell mediated destruction remains clearly complicated. For these reasons, islet regeneration carries a heavy burden. Previous studies have shown that dampening HLA expression could be a potential strategy to escape autoimmunity. While this remains to be seen, there is cautious optimism that this technological step forward will improve patient-centric medicine. As a result, clinical trials are examining gene-editing strategies that enhance stem cells to evade the immune directed rejection. CRISPR Therapeutics are currently involved in an open-label, phase 1/2 study evaluating the efficacy of VCTX211 in people living with T1D (NCT05565248). VCTX211 therapy compromises allogeneic pancreatic endoderm cells by genetic modification using CRISPR/Cas9 to promote immune evasiveness to deliver endoderm cells. Meanwhile, researchers at the University of Chicago are assessing the safety of the monoclonal AT-1501 antibody against CD40 to tone down the immune system and prevent rejection without harming pancreatic islet cells (NCT06305286). Using Treg lymphocytes and the anti-CD20 antibody (Rituximab), a Phase 2 interventional trial led by researchers at PolTREG S.A. will enrol individuals that are at risk of developing T1D. The purpose of that study will be to assess this treatment in the paediatric population to delay T1D onset (NCT06688331). Another study will assess the effectiveness of the humanized monoclonal CD3 antibody Teplizumab in blocking autoimmune rejection in recently diagnosed individuals with T1D (NCT00129259). The study outcomes to date have shown improved C-peptide responses for up to two years without the need for immunosuppressive therapy.

In 1922, insulin proved to be lifesaving for Leonard Thompson, a 14-year-old-boy, who received the first insulin injection to treat T1D at Toronto General Hospital. Today, the landscape for treatment has drastically changed. Medical research is ushering in a transformative era of technology driven in-part by regenerative stem cell therapies, accelerated not only because the incidence of diabetes in young people and adults continues to rise, but also because the need for cure, is deeply personal. New surgical procedures employing transplantation of chemically induced islets from a young adult in Nankai has shown just how far we have come from the first insulin injection. While there is little hesitation in recognising that the advancements in autologous cell therapy are transformative, there is also no doubt this treatment is too early for widespread use - for now – and this will change with advances in technology. In making medical breakthroughs there is often a personal component rarely seen by outsiders. Pointedly, the patient. With all this in mind, showing tolerable safety and insulin injection freedom for 1 year shows just how personal we can be before personalised medicines become more broadly available.
